# Isolated poorly differentiated cancer (insular) in a thyroglossal cyst: a case report

**DOI:** 10.11604/pamj.2021.39.254.30317

**Published:** 2021-08-20

**Authors:** Anas Mohammad Abu Rumman, Majdi Abdelmahdi Alsoudi, Hamzeh Mohammad Qasimeh, Wajdi Abed Alnajada, Ruba Yaseen AL Shunnaq

**Affiliations:** 1 King Hussein Medical Center, King Abdullah II St 230, Amman 11733, Jordan,; 2 Queen Alia Hospital, Amman, Jordan,; 3 Princess Eman Laboratory Center, King Hussein Medical Center, Amman, Jordan

**Keywords:** Insular carcinoma, thyroglossal cyst, thyroglossal duct, case report

## Abstract

A 23-year-old young man presented with a painless neck mass that he noticed slowly growing over the past 4 years. Neck imaging showed an irregular 5 x 5 cm mixed solid and cystic mass in mid neck that looked suspicious but normal looking thyroid and no neck lymphadenopathy. Thyroid uptake scan was within normal also. Fine needle biopsy (FNA) from thyroglossal cyst (TGC) was malignant, Bethesda VI. Multi-disciplinary meeting discussed the case and advised for removal of both the TGC cancer and total thyroidectomy. After patient counseling, he underwent Sistrunk procedure for excision of the TGC mass and total thyroidectomy. histopathological examination revealed a poorly differentiated carcinoma of insular type in TGC with unremarkable thyroid gland. Patient recovery was uneventful. Post-operative multi-disciplinary meeting discussed the histopathology results and advised for post-operative radioactive iodine therapy (RAI) and thyroxine suppression followed by serum thyroid stimulating hormone (TSH) and thyroglobulin (TG). We shall review the diagnostic and management considerations of our case having this rare cancer.

## Introduction

Thyroglossal cyst (TGC) carcinoma has been reported in less than 1% [1] of cases. The most common type being papillary thyroid cancer (75-85%), followed by follicular thyroid cancer and squamous cell [2]. Rarely has the literature reported cases of poorly differentiated cancer in TGC, moreover, no cases of insular type poorly differentiated cancer in TGC was previously reported. Diagnostic and treatment considerations include means of establishing diagnosis, what surgery to plan (i.e.: Sistrunk alone; with or without total thyroidectomy, and which precedes the other), then there are post-operative considerations such as postoperative use of radiotherapy, role for radioactive iodine, role of thyroxine suppression in these cancers, and how to do follow up and by which investigations. The knowledge acquired by managing these very rare cases may be helpful in determining best management practices as well as being the basis for future studies.

## Patient and observation

**Figure 1:**
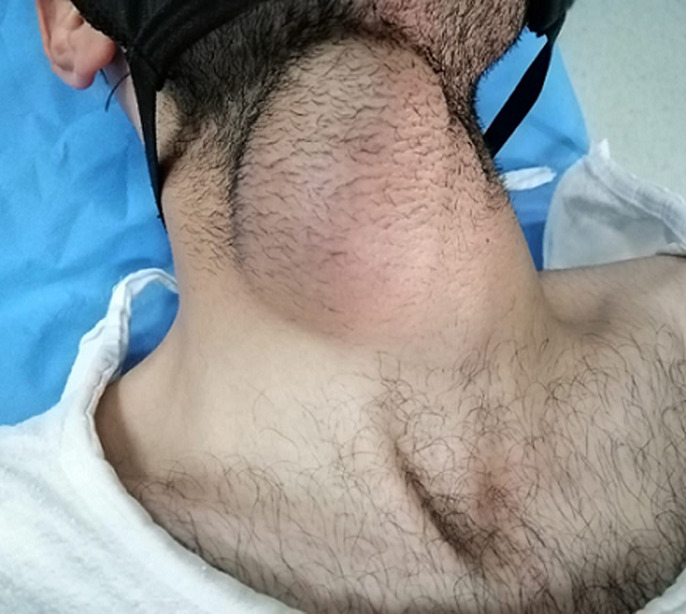
clinical presentation

**Patient information:** a 23-year-old young man, healthy and not known to have any prior medical illnesses was referred to our clinic complaining of a painless neck mass that he noticed slowly growing for the past 4 years (Figure 1). The mass enlarged dramatically over the last 3 months and became obvious. It also started to cause difficulty in swallowing and shortness of breath. There were no symptoms of fever, chills and rigors, pain on swallowing, loss of weight or appetite nor any other masses in the neck. He had no family history of thyroid cancer or any neck malignancies.

**Clinical findings:** on examination, vital signs were normal. There was a large central mass in the mid-neck, measuring 4 x 5 cm, oval, irregular in shape, hard in consistency and non-tender. There were no overlying skin changes and no intraoral extension. The mass moved slightly with tongue protrusion and deglutination but was difficult to assess due to its large size. There were no palpable cervical lymph nodes.

**Timeline:** the patient presented with painless neck mass in mid April 2021, clinical, radiological, and pathological assessment began immediately. Sistrunk procedure performed in 26^th^ April 2021, which revealed isolated poorly differentiated cancer (insular) in a thyroglossal cyst, multidisciplinary team (MDT) decision was to proceed for total thyroidectomy which done after 3 weeks of the first surgery, patient recovered uneventfully and discharged home four days after surgery. Then the patient sent for nuclear medicine and endocrinologist to continue his treatment, where he received radioactive iodine in the nuclear medicine department one month after the second surgery and thyroxin replacement treatment by the endocrinologist.

### Diagnostic assessment

**Blood workup:** hemoglobin level was 15.6 g/dL. Total white cell count was 10.5 x 10^9^/L. Serum C reactive protein and procalcitonin levels were normal. Thyroid screen was normal with free T4 at 17.86 pmol/L and thyroid-stimulating hormone (TSH) at 0.975 mIU/L. Kidney function test, liver function test and coagulability test were within normal levels.

**Radiological workup:** neck ultrasound: 5 x 5 cm irregular cystic mass with solid component with increased vascular flow over the solid component in ant neck, no lymph node enlargement in neck. The thyroid looks normal, no enlargement and no nodules seen. Thyroid uptake scan: normal uptake in both thyroid lobes, no cold nodules. Neck magnetic resonance imaging (MRI): showed irregular looking mixed cystic and solid soft tissue mass anterior to the hyoid bone measuring 5 x 5 cm compressing the trachea, no nodules in both thyroid lobes, and no enlarged lymph nodes in the neck.

**Diagnosis:** the case was discussed at a multidisciplinary meeting with representatives from surgery, pathology, radiology, endocrine and oncology. Benign differentials included TGC with atypia, ectopic thyroid nodule, and lymphangioma, but the irregularity and vascularity over the solid component on ultrasound was in favor of a malignant process. Ultrasound guided fine needle aspiration (FNA) of the solid component of the mass was performed and the result was malignant, Bethesda category VI, with features suggestive of papillary thyroid carcinoma.

**Figure 2:**
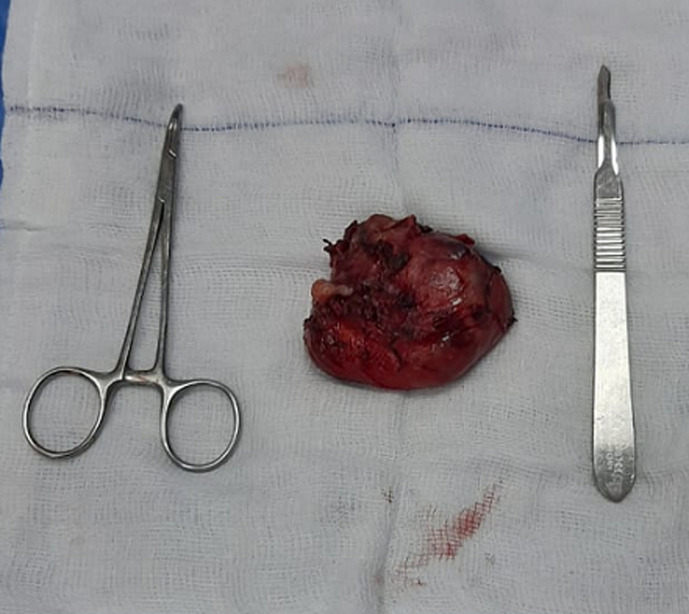
thyroglossal cyst

**Therapeutic intervention:** Sistrunk operation was first performed with en bloc resection of the mass with surrounding involved muscle tissue and mid-section of the hyoid bone (Figure 2). Then total thyroidectomy was performed. Two peri-thyroid enlarged lymph nodes were seen and were excised and sent for frozen section along with frozen section of the thyroidectomy specimen. The thyroid specimen showed no evidence of carcinoma and the 2 lymph nodes were reactive. During surgery, four parathyroid glands were identified and preserved and the recurrent laryngeal nerves were identified and preserved bilaterally.

**Figure 3:**
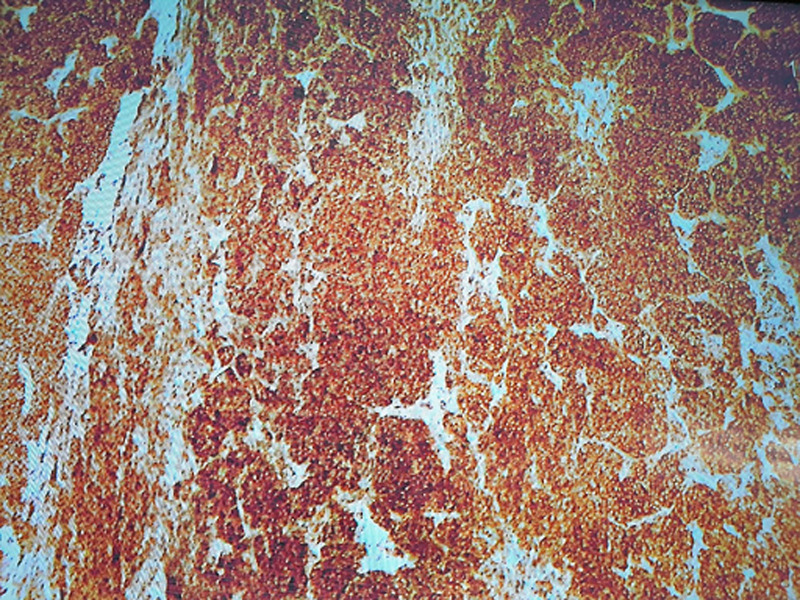
positive immunostain

**Figure 4:**
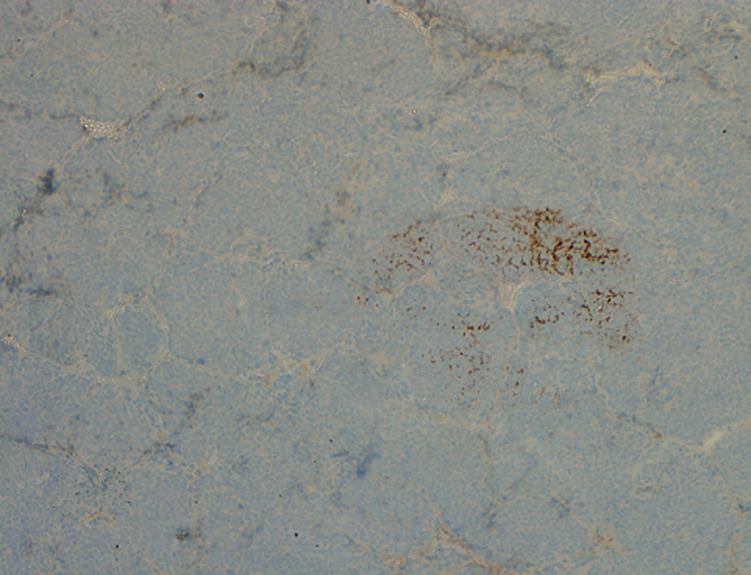
thyroglobulin immunostain

**Figure 5:**
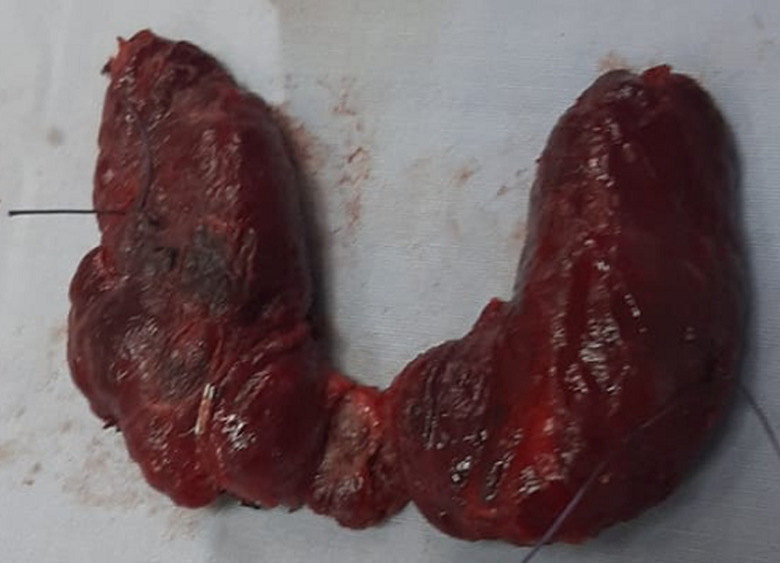
bilateral thyroid specimen

**Follow up and outcome:** the patient had an unremarkable recovery period and was discharged well on postoperative day 4. He was given calcium tablets and analgesia. Histopathology showed poorly differentiated (insular type) carcinoma arising from TGC with surrounding skeletal muscle involvement. The maximum dimension was 7cm. The tumor was composed of small uniform hyperchromatic cells with convoluted nuclei arranged in solid, insular and trabecular patterns. There was tumor necrosis, mitotic figure was 40MF/10HPF, multiple foci of vascular space invasion, no nuclear features of papillary carcinoma was seen, cyst wall containing focus or normal thyroid follicles. Immunohistochemical staining (IHC) stains used: thyroglobulin focally positive, B-cell lymphoma-2 (BCL_2_): positive, creatine kinase (CK): positive, protein 53 gene (P53): focally positive, focally positive, cytokeratin 19 (CK19) and epithelial membrane antigen (EMA) were positive but synaptophysin, chromogranin, calcitonin, high molecular weight cytokeratin (HMWCK), thyroid transcription factor 1 (TTF-1), neural\melanocytic marker (S100), leukocyte common antigen (LCA), Desmin, paired box gene-8 (PAX-8), creatin kinase-7 (CK7) were all negative (Figure 3, Figure 4). Total thyroidectomy specimen showed benign thyroid tissue with no tumor identified (Figure 5). The two peri-thyroid lymph node (LN) were reactive. The resection margins were all clear.

## Discussion

During embryogenesis the thyroid may stop its descent from the base of the tongue till its normal site over the second, third and fourth tracheal rings [3,4], forming ectopic thyroid. Ectopic thyroid is defined as thyroid tissue not located anterolaterally to the second and fourth tracheal cartilage [5]. The most common site is the thyroglossal duct which contains functional thyroid follicular cells in the cyst wall in more than half of TGCs [6]. TGC is the most common central neck mass in pediatric patients [7-9] presence of a palpable, painless, slow-growing, midline neck mass is a common presentation. Treatment of TGC is by complete excision of the cyst and the thyroglossal duct with central hyoidectomy [10]. The limitation in number of cases with Thyroid cancer in TGC resulted in differences in the treatment of TGC cancer. Major controversy regarding the need for total thyroidectomy in the course of treatment of TGC malignancy [1,11], the need for lymph nodes dissection, and how often to do follow up after. A recent review of thyroid cancers arising in ectopic thyroid tissue in the neck concluded that each case should be managed individually.

Poorly differentiated thyroid carcinomas (PDTC) behavior and histologic features are intermediate between well-differentiated and undifferentiated thyroid carcinomas [12]. One of the known PDTC is insular carcinoma. The frequency of this tumor appears to be higher in central Italy than in The United States and other countries with a slight female predominance. Grossly, the tumors appear solid, gray-white with foci of necrosis and infiltrative margins [13]. At presentation, regional metastases in around one third of cases and distant metastases reported in one fourth of cases was observed in several series of cases [14].

In our case, histologically, the tumor was composed of small uniform hyperchromatic cells with convoluted nuclei arranged in solid, insular and trabecular patterns. No nuclear features of papillary thyroid carcinoma were seen. The tumor had frequent mitotic figures and there were foci of necrosis in addition to presence of both vascular and lymphatic invasion. All these features are compatible with PDTC. The immunohistochemical stains were used to exclude other probable differential diagnoses such as medullary thyroid carcinoma and paraganglioma, confirmed by negativity for calcitonin, synaptophsin and chromogranin immunostains respectively. Poorly differentiated carcinoma was favored over undifferentiated carcinoma by strong positivity for BCL_2_ and focal positivity for p53 immunostains. Other differential diagnoses were excluded including melanoma, lymphoma, rhabdomyosarcoma and squamous cell carcinoma by negativity for S100, LCA, Desmin, and high-molecular-weight kininogen (HMWK) respectively. Thyroid transcription factor 1 (TTF-1) is usually positive in poorly differentiated thyroid cancer (87%) of cases, but in our case it was negative. In view of the histologic features, positivity for BCL_2_, thyroglobulin and exclusion of other differential diagnosis, the most compatible diagnosis was insular type poorly differentiated thyroid carcinoma arising in a TGC. There is no clear consensus on treatment modalities for insular carcinomas but for those tumors with a pure insular morphology, treatment options is similar to those used for undifferentiated carcinomas. Metastases in these cancers are common, with the most common sites being lymph nodes (regional), followed by liver then bone, thus prognosis is poor.

In the current presented case, our patient is a young male who presented with large painless anterior neck swelling of 4 years duration that he noticed to have enlarged recently and has started to cause compressive symptoms. Ultrasound showed a suspicious irregular shaped 5 x 5 cm cystic and solid mass without signs of cervical lymphadenopathy, FNA under ultrasound guidance confirmed presence of malignant cells. Surgical intervention was performed which included a Sistrunk procedure in addition to total thyroidectomy. While some authors consider Sistrunk´s procedure to be adequate and curative in most cases, others recommend that total thyroidectomy should be performed in case of TGC carcinoma, due to the high incidence of associated papillary or mixed carcinomas in the thyroid gland [15], and its removal aids staging and can facilitate the detection of metastases and follow up with thyroglobulin.

## Conclusion

Thyroglossal cyst carcinoma is rare, but should be thought of as a differential in patients with an enlarging midline neck mass especially if radiographical features are in favor, ultrasound guided FNA cytology is a simple, minimally invasive and very important in establishing diagnosis, the addition of total thyroidectomy with no evidence of malignancy should be discussed with the patient, weighing the risks and benefits, as some patients with isolated TGC cancers were reported of having thyroid cancer years later, neck MRI is beneficial in poorly differentiated cancer because it can pre-op detect surrounding soft tissue involvement.
